# Workflow for Maxilla/Mandible Individual [Mai^®^] Implant by Integra Implants—How Individual Implants Are Manufactured

**DOI:** 10.3390/biomedicines12081773

**Published:** 2024-08-06

**Authors:** Rafał Zieliński, Agata Kołkowska, Jerzy Sowiński, Bartłomiej Konieczny, Marcin Kozakiewicz, Wojciech Simka

**Affiliations:** 1 Stomatologia na Ksiezym Mlynie, Lodz, 16D Tymienieckiego, 90-365 Lodz, Poland; 2 Department of Inorganic Chemistry, Analytical Chemistry and Electrochemistry, Faculty of Chemistry, Silesian University of Technology, 44-100 Gliwice, Poland; agatkol653@student.polsl.pl; 3 Chemistry Students Research Society, Faculty of Chemistry, Silesian University of Technology, 44-100 Gliwice, Poland; 4 Private Dental Clinic, Tetmajera 3A Rd., 05-080 Izabelin C, Poland; jersow@gmail.com; 5 University Laboratory of Materials Research, Medical University of Lodz, Pomorska Str. 251, 92-213 Lodz, Poland; bartlomiej.konieczny@umed.lodz.pl; 6 Department of Maxillofacial Surgery, Medical University of Lodz, Żeromskiego Str. 113, 90-549 Lodz, Poland; marcin.kozakiewicz@umed.lodz.pl

**Keywords:** individual implant, subperiosteal implant for maxillofacial, Mai Implants^®^, technology process of manufacturing individual implants, subperiosteal implants

## Abstract

The newest technology allows the medical industry to manufacture innovative products such as milled titanium prosthodontic parts in an implant for a screw-retained suprastructure. In the literature, there are some articles on the clinical usage of subperiosteal implants, but none of these publications, either in PubMed or Google Scholar, thoroughly describe the workflow for the design and manufacture of individual implants for maxillofacial surgery with milled threads for a screw-retained prosthodontic bridge. The aim of the article is to present a step-by-step method of producing personalized implants, from the first steps of production to the implantation of the final product. The article includes information on patient qualification for surgery, computational preparation and skull printing, planning of Mai Implants^®^, meshing, 3D printing and milling, cleaning, rinsing, anodizing, and laser marking, as well as the cleaning and sterilization process in a hospital or dental clinic. A detailed description of implant production allows for the analysis of each step and the development of technology. The production of implants is an expensive procedure, but considering all the advantages of the Mai Implants^®^ treatment and the disadvantages of alternatives, the product is worth the price.

## 1. Introduction

Sub-periosteal implants gained popularity during the 1950s and 1960s but fell into disuse with the advent of endosseous titanium implants. The decline in their use was due to several factors. Initially made from Vitallium, an alloy of cobalt, chromium, and molybdenum, using the lost-wax technique, these implants had a mismatch in elasticity with bone, leading to stress shielding and implant fixation issues. Additionally, Vitallium’s inability to integrate with soft tissue or bone made it susceptible to infections, which often resulted in significant bone loss and fistula formation extending into the nose and sinus cavity.

Similar complications are observed today in cases of uncontrolled peri-implantitis in bone-grafted maxillae. However, recent advancements have introduced 3D printed titanium sub-periosteal implants as a solution for these challenging cases. Previously, the process required two separate surgical procedures: one for obtaining impressions and another for implantation, typically without screw fixation. The discovery of titanium’s suitability as an implant material revolutionized oral endosseous implant production [1,2].

Clinicians have employed various strategies to address severe bone atrophy, including implantation in distant bone areas such as the zygoma, local and distant bone augmentation, osseodistraction, and guided bone regeneration. These methods aimed to connect endosseous fixtures with suprastructures, which became the gold standard. Zygoma implants, boasting a clinical survival rate of 96.7%, can lead to complications such as sinusitis, soft tissue infections, and oronasal fistulas, and present technical challenges for prosthodontists and laboratory technicians [3].

In patients with Cawood and Howell classes V–VI, successful long-term intraosseous dental implants are unachievable without extensive bone grafting, which is often sourced from the anterior iliac crest or calvarial bone [4,5,6]. Alternatively, zygomatic implants can be used for edentulous patients with atrophied maxillae, though they carry a high risk of complications including prosthesis failure, zygomatic implant fracture, paresthesia/dysesthesia, oro-antral communication, sinusitis, apex infections, peri-implant mucositis, peri-implantitis, and tissue retraction or orbital cavity penetration [7,8,9,10].

Based on our three-year patient follow-up, we have found that these complications are nearly nonexistent in titanium subperiosteal implants when an appropriate surface and well-planned shape are prepared. The newest technology allows for the manufacturing of innovative products such as milled titanium prosthodontic parts in an implant for a screw-retained suprastructure. While some literature discusses the clinical usage of subperiosteal implants, none thoroughly describes the workflow for designing and manufacturing individual implants for maxillofacial surgery with milled threads for a screw-retained prosthodontic bridge.

This article aims to present the step-by-step manufacturing process of subperiosteal implants using selective laser melting (SLM) to produce a prosthodontic restoration with the advanced Maxilla/Mandible Individual [Mai^®^] implant by the Polish company Integra Implants^®^.

## 2. Materials and Methods

### 2.1. Patients’ Qualification for the Operation

The requirements regarding the general health of patients who are potential candidates for subperiosteal implants are similar to those for traditional dental implants [8]. The main difference that influences the choice of the solution used is bone loss. Individual implants are performed when the alveolar ridge of the jaw is too thin and too low, both in the posterior and anterior sections [11]. According to Cawood and Howell, Mai Implants^®^ class implants should be used in classes V and VI when the alveolar bone is completely resorbed.

**Figure 1:**
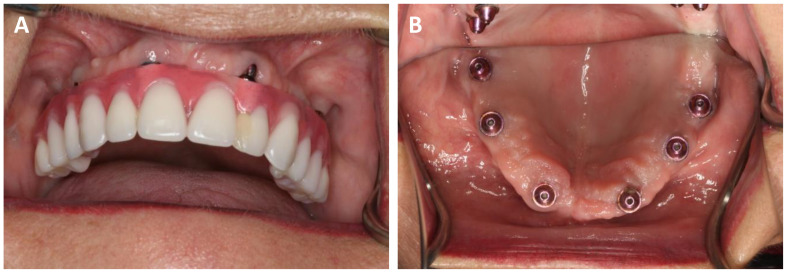
(**A**) Composite visible on the vestibular surface on the tooth. (**B**) The same patient without a bridge. The excessively vestibular setting of the multiunit has resulted in anunesthetic hole that must be covered with composite on the acrylic bridge in a dental clinic.

After admission to the hospital, the patient must undergo cone beam computed tomography (CBCT) or head computed tomography with appropriate parameters [Table 1]. It is optional but helpful to perform another CBCT examination using a prosthetic restoration with radio-opaque gutta-percha balls embedded in a removable denture to bypass the vestibular holes for a screw-retained bridge [Figure 1]. If practitioners decide on CBCT with a removable prosthesis, a 3D scan of the removable prosthesis is mandatory for the superimposition of the .STL files.

The most important factor for all implants—intraosseous, zygomatic, and Mai Implants^®^—is the thickness of the keratinized mucosa around the implant/dental abutment and its apical edge. Additionally, the relationship of the upper and lower jaws to possible future overbite in dental implant bridges must be taken into account.

### 2.2. Computational Preparing and Printing of a Skull

**Figure 2:**
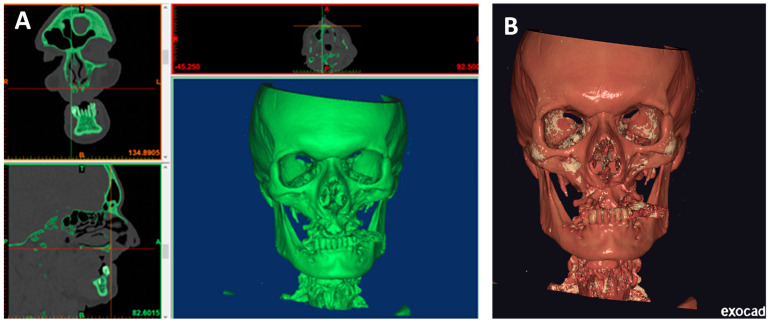
(**A**) Setting the threshold in the software for segmentation purposes—Materialise^®^ (upper image—front view; bottom image—lateral view). (**B**) Importing .DICOMs and .STL files into Exoplan^®.^

After sending the CT/CBCT scan results to the manufacturing center, a bioengineer must import the files into software for segmentation purposes [Amira^®^ v2 by Thermo Fisher Scientific Inc., Waltham, MA, USA or Mimics Medical^®^ v2 by Materialise, Leuven, Belgium or others]. The first step in segmentation is importing the .DICOMs into the software and setting the appropriate threshold for dividing the bone [Figure 2A]. The model consists of an .STL file, and .DICOMs are imported to Exoplan^®^ [ExoCAD GmbH, Darmstadt, Germany] [Figure 2B].

**Figure 3:**
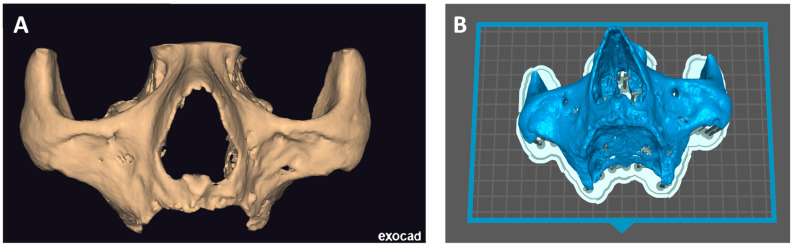
(**A**) STL file after surface polishing in GeoMagic Studio^®^ imported into ExoCAD^®^and ready for export into 3D printer software. (**B**) Skull model with suprastructures set in 3D printer software.

In Exoplan, the .STL file is edited—unnecessary material, artifacts, and lower jaw are cut off, and holes are filled. Then, the file is saved and transferred to other software [GeoMagic Studio^®^ v2 by Artec Europe or MeshMixer^®^ v2 by Autodesk] for surface polishing [Figure 3A]. Such a prepared file is ready for printing [Figure 3B]. Perfect Mai Implant^®^ fit-ting to the bone is crucial; thus, the best quality 3D printing technology is of utmost importance. Such precision can be obtained on a high-resolution printer: multijet printing (MJP) technology, for instance, on ProJet 2500 Plus [3D Systems, Inc., 333 3 D Systems Circle, Rock Hill, SC 29730, USA]. In this technology, selective MJP is an inkjet printing process that uses piezo print head technology to deposit photo-curable plastic resin.

### 2.3. Mai Implant^®^ Design

**Figure 4:**
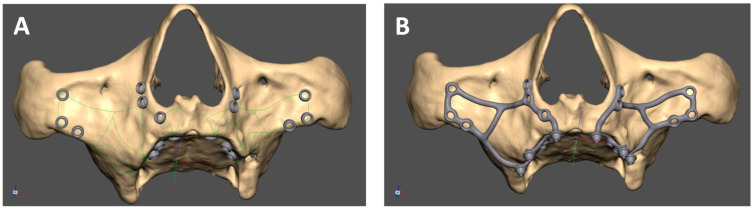
(**A**) Mai Implant^®^ with holes where the bone is sufficiently thick. (**B**) Holes linked with connectors and the placement of multiunits in Mai Implant^®.^

The manufacturing center gets in contact with the practitioner with a view to designing the Mai Implant^®^. The first thing the bioengineer does is import and analyze the bone model in an .STL file in Geomagic Freeform Plus^®^. Then, the implant mesh is designed in an .STL file. Holes for screws in areas where the bone is sufficiently thick are designed. After the modeling of implant meshes and placement of multiunits in appropriate positions, finishing, contouring, and adjustments of the implant to the bone are performed [Figure 4]. Then, an .STL file of the Mai Implants^®^ is exported. 

**Figure 5:**
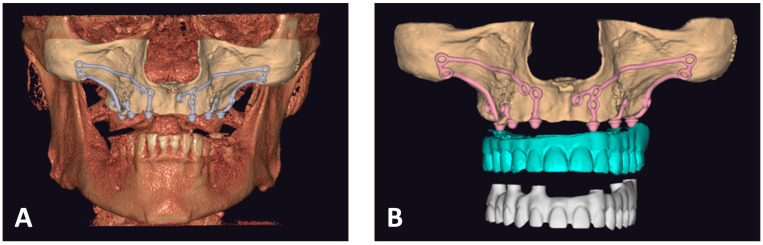
(**A**) An individual implant after the segmentation process. (**B**) The Mai Implant^®^ after the superimposition of a CBCT scan image with a removable prosthesis with radiopaque gutta-percha balls.

A cone-beam computed tomography scan performed with a prosthesis with radiopaque/gutta-percha balls is recommended to plan a multiunit channel on the occlusal/palatal surface; however, the multiunit must always sit on the alveolar bone on the maxilla [Figure 5].

### 2.4. Manufacturing 

#### 2.4.1. Mesh Creation

**Figure 6:**
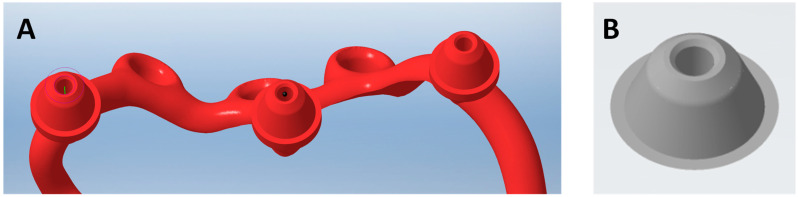
(**A**) Internal threads in the multiunit. (**B**) The Integra Multiunit^®^.2.5 project (**B**).

In iCAM v5 Smart [iMes iCore], the Mai Implant^®^ design is set in a frame. Small black holes indicate internal threads in the multiunit, and then the interface is changed to the original Integra Multiunit^®^ [Figure 6]. 

**Figure 7:**
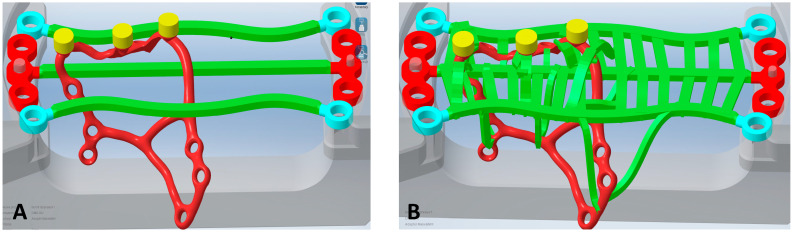
(**A**) The mesh with three main connectors. (**B**) The mesh with new connectors.

To hold the implant firmly in the mesh, three main connectors are designed to run across the frame [Figure 7A]. New connectors are then designed in such a way that all small connectors are attached to the already-designed ones [Figure 7B]. The reason for that is to minimize the risk of movement of the Mai Implant^®^ during milling and to make a fixed mesh of titanium arms. As a control, all the connectors are double-checked to ensure they are un-broken and attached to the Mai Implant^®^. The file is saved in a folder. Then, a new program is generated for the milling machine [DMG Ultrasonic 20, DMG Mori, 2-3-23 Shiomi Koto-ku, Tokyo, 135-0052, Japan] operator for milling the multiunits.

#### 2.4.2. 3D Printing and Milling

On a Lasertec 12 SLM machine, the file is imported into CAMbridge^®^ v2 and Celos^®^ v2 software [DMG Mori, 2-3-23 Shiomi Koto-ku, Tokyo, 135-0052, Japan]. The machine’s container is filled up with titanium powder [Starbond Ti5, Scheftner^®^]. The mesh with implants is placed on a virtual base plate in CAMbridge^®^. In the program, the mesh is positioned, and supports are designed. The whole base plate is divided into many slices, or layers, every 30 µm. Then, in R-designer^®^, all the laser parameters are set for use during the melting process. After the procedure, the implant with the mesh and the base plate undergoes a thermal process to enhance the parameters of titanium and remove stress during melting. The whole process takes about 12 h.

The titanium 3D printer laser beam melts titanium powder layer by layer [30 µm]. It is very important to validate the powder regularly and maintain its quality during the processes to prevent, in the next melting process, the occurrence of any conglomerates of powder that appeared during the previous process [12]. Lasertec 12 printer has a closed circuit, so oxygen does not have contact with powder before opening the door. Powder is cleaned using a vacuum cleaner and moved into a separate storage. The route to the storage is automatically closed off tightly to prevent contact with oxygen. 

After finishing the process, the base plate with implants is moved to the vacuum furnace [Amazemet^®^, Jana Pawła II 27, 00-867 Warsaw, Poland]. Then, the whole structure is cut off, and supports are gently removed through drills. It is important not to use scissors in order not to bend the structure.

**Figure 8:**
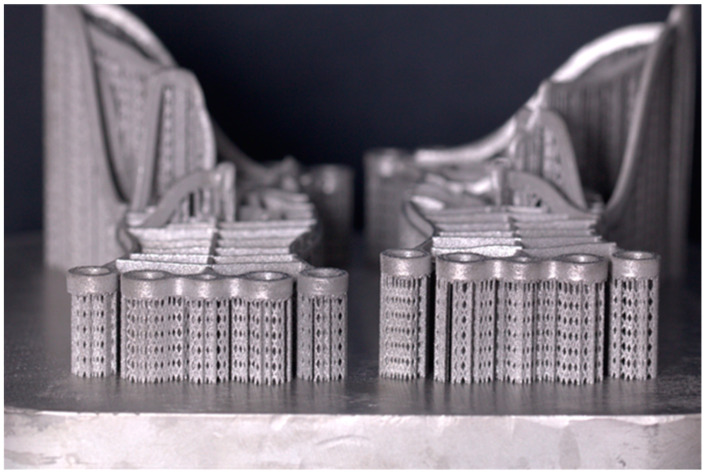
Three-dimensional printed Mai Implants^®^ just before removal from the Lasertec 12 SLM (DMG Mori, 2-3-23 Shiomi Koto-ku, Tokyo, 135-0052, Japan).

The next step is to put the implant in a milling machine DMG 20 to mill the internal threads. Then, the Mai Implants^®^ are hand-adjusted onto the 3D printed model of the bone using drills. Surfaces in contact with the bone are sand-blasted with Al_2_O_3_, and those in contact with gingivae are gum-polished [Figure 8].

#### 2.4.3. Cleaning, Rinsing, Anodizing and Laser Marking

Cleaning and rinsing are crucial to remove the residuals after processing. The first cleaning is performed in a stainless steel bucket at a frequency of 27 kHz for 5 min, then for another 5 min at a frequency of 80 kHz. The first bucket consists of two containers. The first is a washer, and the second is an oil separator. At a frequency of 27 kHz, cavitation bubbles have greater energy, thanks to which they can eliminate stubborn dirt, while 80 kHz allows the elimination of dirt in small holes and from porous surfaces. The workpiece is then rinsed twice, using detergent and water in reverse osmosis (RO) with and without ultrasound.

**Figure 9:**
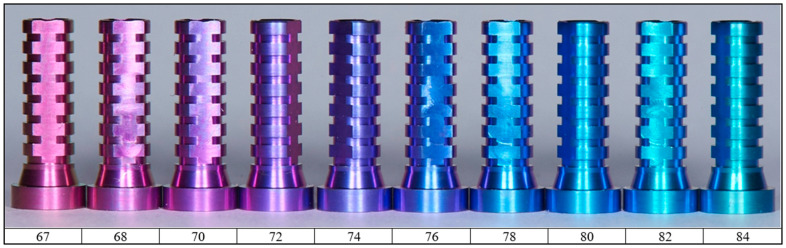
Example of Ti6Al4V samples anodized in different voltage; numbers indicate an anodizing voltage [V].

The final stage is anodizing the detail. The most desirable color is pink because of its similarity to the color of the gums. This color makes the implant less visible, especially compared to a classic metal implant. The shade of the obtained coating depends on the conditions of the anodizing process, which influence the different thicknesses of the layer [13]. The dependence of the obtained color on the voltage used for anodizing is shown in [Figure 9]. Regardless of the electrical voltage, the current rise time and the voltage application time must be constant. Based on the authors’ experience, the parameters of the anodizing process were selected [14]. So, the anodizing was conducted in a 98% H_3_PO_4_ solution.

**Figure 10:**
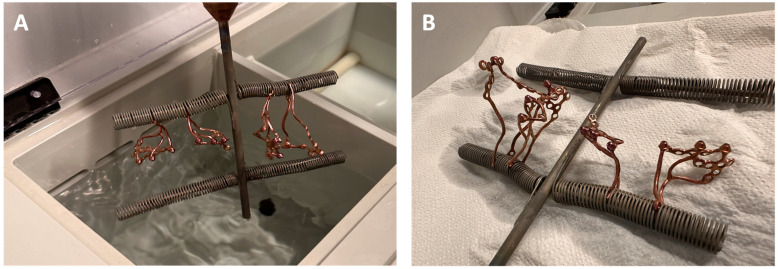
Anodizing process—individual implants mounted to an anodic holder: (**A**) In an electrolyzer; (**B**) Drying on air.

The mounting of implants during the anodizing process is shown in [Figure 10]. 

**Figure 11:**
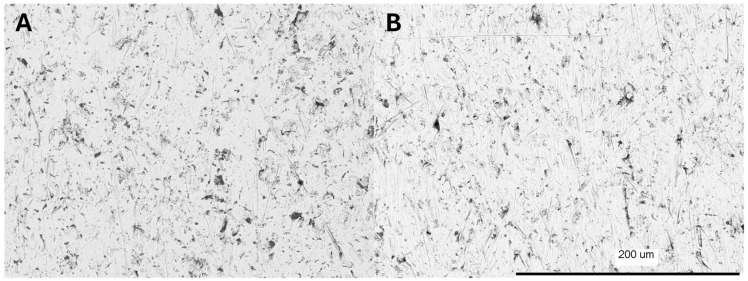
SEM pictures of rough Ti6Al4V surface (**A**) and anodized surface (**B**).

Electrochemical processes lead to characteristic surface modification. For anodized implants, there is a huge difference in surface characteristics, which can be seen by scanning electron microscope (SEM). The morphology of the implant surface strongly depends on the applied voltage. In the case of voltages higher than about 95 V, a porous surface is obtained, and the plasma electrochemical oxidation process takes place. Below this voltage, the oxide layer formed in the anodizing process reflects the substrate and is smooth [14]. In the case of Maja Implants^®^, the process voltage was selected in such a way as to obtain a pink color of the oxide layer and, thus, of the entire element [14]. The figure [Figure 11] shows the morphology of the implant surface before and after anodizing. According to the literature data, no changes in the surface morphology were noted after the anodic dyeing process [15]. 

After the anodizing process is completed, the pink implants are put on a laser marking machine [TruMark Station 5000+, Trumpf 111 Hyde Road, Farmington, CT 06032, USA]. An engraving is made on one of the arches of the implant showing the patient number, in compliance with the data protection laws, and the side of the maxilla—left as “L” or right as “R”—to inform the surgeon which implant they should use during the operation.

### 2.5. Cleaning and Sterilization Process in a Hospital or Dental Clinic

After receiving the implant packed as a non-sterile product at a dental clinic or hospital, the practitioner is responsible for performing appropriate cleaning and sterilization in the autoclave process. Each implant is delivered in a clean but not sterile disposable bag, along with the order number and a label for sterilization. The whole process of cleaning, disinfection, and sterilization must be performed under the principles approved by the hospital or clinic. 

After removing the implant from its packaging, any surface contamination (resulting, for example, from damage to individual packaging) should be removed using disposable wipes or brushes made of synthetic material (nylon brushes are recommended). Only the cleaning and disinfection agents that are suitable and approved for use with medical devices should be selected. If possible, ultrasonic cleaning should be used. Implants should be completely immersed in a cleaning solution. The exposure time of the cleaning agent must follow the manufacturer’s recommendations; individual components during cleaning should not come into contact with each other. If the ultrasonic device does not have a rinsing and drying chamber, the implants must be thoroughly rinsed with demineralized water. The procedure of preparing implants before use involves cleaning in a cleaning solution (e.g., EffectiveInstruExtra^®^) and, after that, in demineralized water at 70 °C for 30 min.

Effective cleaning and disinfection are essential for successful sterilization. Once the implant is cleaned, disinfected, and dried, it can undergo the sterilization process according to the procedures applicable to the user of the product (in the unit performing the implantation) [16].

The recommended method of sterilization is steam sterilization. All non-sterile products must be steam sterilized in an autoclave which is extensively described in the standards EN 1360 and EN 285 [17,18]. The following parameters for the steam sterilization process have been set by the requirements of the established sterilization standards [Table 2].

To avoid damaging the implants during sterilization, individual components should be separated from each other. The sterilization process must be properly planned, carried out, and validated in accordance with applicable standards and procedures. Sterile implants meet the requirements of EN 556-1 [19] for product sterility at a Sterility Assurance Level (SAL) of 10-6. Sterilization should be carried out in accordance with standard procedures. The sterilization process has been validated in accordance with the recommendations of ISO 17665-1 [20].

Non-sterile implants cannot be sterilized in the packaging in which they were delivered. The product should be placed in a double-layered paper-foil pouch according to ISO 11607-1 [21], intended for steam sterilization in accordance with the requirements of ISO 17665-1 [20].

All the manufacturing processes have been validated and approved by TÜV Rheinland.

## 3. Discussion

Three-dimensional printing technology is a tool that is developing very quickly in implantology. Newer and more precise devices make it possible to produce individual implants with complex shapes adjusted to the patient [22,23]. This is especially important in the case of maxillofacial surgery. This article introduces a prosthesis-driven, reverse-engineering approach that employs CAD/CAM (computer-aided design and computer-aided manufacturing) and additive manufacturing to restore both function and aesthetics in a single surgical session, occasionally using only local anesthesia. This technique may also be of benefit in cases of full or partial edentulism and post-resection defects. In 100% of the author’s cases, an immediate temporary bridge was delivered within a maximum of 72 h after the surgery. The bridges were made from acryl reinforced with metal wire. Additional costs may occur when patients’ aesthetic expectations are not met or when extensive occlusal adjustments are needed. Dental laboratories should have experience when it comes to making temporary bridges similar to those used in All-on-4 cases. 

Despite its advantages, the potential for peri-implantitis remains a concern with this technique. Peri-abutment mucositis can develop, which can be managed by disconnecting the abutment from the main frame through the use of high-speed rotating instruments. The design of Mai Implants^®^ allows the practitioner to cut off any connectors that are causing dehiscence. Thus, the number of multiunits is always designed and manufactured in excess. Concerning peri-implantitis, the situation will resemble that of zygoma implants, in which fixation at the zygomatic end of the Mai Implant^®^ remains unaffected. In many cases, insufficient hygiene or insufficient sealing between the implant and bridge might be the reason for dehiscence. However, the manufacturer of Mai Implants^®^ ensures that the gap between the milled suprastructure in the company’s milling center and the multiunit on the implant is approximately 20 µm, so the risk of inflammation because of bacteria plaque is reasonably low. From the author’s experience, proper fitting of Mai Implants^®^ to the bone is a key factor of long-term success. Thus, it is crucial to use the highest-precision 3D printer.

The use of anodizing as a method to improve aesthetics greatly increases the possibilities of selecting an individual implant. Reduced implant visibility due to the use of a color similar to the color of the gums is an undeniable advantage of Mai Implants^®^ [24]. Anodizing also affects the most important factor during implantation—osseointegration. A good connection between the tissue and the implant is the key to a successful implantation procedure. Anodizing can create permanent bonds with the tissue, leading to a stable embedding of the implant in the facial bones for years [25,26]. The anodizing process can also improve the biocompatibility of dental implants by changing their surface. By stimulating the formation of metal oxides, anodized implants can reduce the risk of immune reactions. By improving tissue biocompatibility and adhesion, anodized implants can accelerate the healing process after implantation, shortening recovery time and reducing the risk of postoperative complications [27,28]. In addition to the enormous advantages in the biological area, the above-mentioned surface treatment also affects the mechanical properties of the implants. Anodizing creates a hard protective coating on the implant surface, which protects it against mechanical damage, such as scratches or abrasion, which may be particularly important in the oral cavity, where implants are exposed to food and other substances. Additionally, this coating protects the more exposed shaft from corrosion in the aggressive environment of the oral cavity [29,30,31]. New trends indicate the possibility of further surface modifications.The titanium anodized surface significantly increases blood clot retention, significantly increases nano-roughness, and favors osseointegration [32,33]. Additionally, incorporating marine polysaccharides into drug delivery and tissue engineering has shown significant promise due to their biocompatibility, biodegradability, and ability to form hydrogels. These properties have led to their vast use in developing innovative drug delivery systems and tissue engineering applications. Marine sulfated polysaccharides like carrageenan, ulvan, and fucoidan, extracted from macroalgae, have demonstrated numerous biological properties, including antioxidant, anticoagulant, anticancer, antiviral, anti-inflammatory, and tissue regenerative capabilities. Their ability to form various carriers, such as nanoparticles, hydrogels, and scaffolds, makes them suitable for controlled drug release and enhancing tissue regeneration [34].

Despite the fact that Mai Implants’^®^ workflow is the same for the jaw because of thinner and moveable mucosa, the risk of periimplantitis is higher than in the maxilla [35].

The authors have a promising 3 years of clinical experience as described in a different publication. The following article is a technical note. It is not a clinical article. The article with clinical data is a separate scientific work that we are in the process of publishing. However, it is worth noting that complications after Mai Implants^®^ were as follows:

(A) Multiunits movements—6%;
(B) Implants exposure—9%;
(C) Recurrent swelling—5%.

Implant loss 3 years after surgery was 3%, so it is comparable to the success rate of zygomatic implants or conventional implants [36]. Implant loss was caused in patients treated at the beginning of the whole group of 80 patients.

## 4. Conclusions

The search for a different method of producing implants is a development path due to the multitude of possibilities offered by the use of various production parameters, including implant surface treatment. Moreover, 3D-printed subperiosteal implants are a great alternative to classic implants, and their improvement is an area of research worth paying attention to. This paper presents the workflow of individual implant production, which can be developed in other research centers or companies. The solutions developed by the authors allowed the production of very good quality Maxilla/Mandible Individual implants. These implants have been successfully used in clinical practice.

## Figures and Tables

**Table 1:** Parameters required for a CBCT scan for a Mai Implant^®^.

Description	Requirements
Time of a scan	Not older than 3 months
Rows × pixels	512 × 512
Tube voltage	90–120 kVp
Slice thickness for CT	0.5–0.7 mm
Slice thickness for CBCT	0.15 mm
Feed per rotation	Max. 1.0 mm
Reconstructed slice increment	Max. 1.0 mm
Reconstruction algorithm	High-resolution bone
File extension	DICOM [Digital Imaging and Communication in Medicine]
Compression	Uncompressed
Optional	Overlapping axial slices
Patient preparation	Remove any non-permanent metal prostheses (if possible, also permanent) and other types of extracorporeal metal (jewelry). Ensure adequate head immobilization and instruct the patient not to swallow during examinations, especially during CBCT, and to remain motionless. The patient may not appear during the entire examination.

**Table 2:** Parameters for steam sterilization of a Mai Implant^®^.

Temperature	134 °C
Exposition time	≥4 min
Drying time	>20–30 min

## Data Availability

The data presented in this study are available on request from the corresponding author. The data are not publicly available due to company confidentiality.
